# Progressive multifocal leukoencephalopathy as the initial manifestation of relapsed lymphoma: a case report

**DOI:** 10.3389/fonc.2026.1710057

**Published:** 2026-02-18

**Authors:** Shi Chen, Haitian Nan, Junjie Li

**Affiliations:** 1 Department of Neurology, Xuanwu Hospital, Capital Medical University, Beijing, China; 2 Department of Neurology, Chifeng Municipal Hospital, Chifeng, China

**Keywords:** case report, JC polyomavirus, lymphoma, PML, progressive multifocal leukoencephalopathy, relapse

## Abstract

Progressive multifocal leukoencephalopathy (PML) is a rare and often fatal demyelinating disease of the central nervous system (CNS) caused by the reactivation of the JC polyomavirus (JCPyV). While PML is a known complication of hematological malignancies, it is exceedingly rare for it to manifest as the initial neurological symptom of a relapsed lymphoma, occurring even before the start of new systemic chemotherapy. We present a case of a 22-year-old male with a history of lymphoma who presented with progressive neurological deficits. The patient’s symptoms, including right-sided hemiparesis and aphasia, were initially mistaken for a CNS lymphoma relapse. Subsequent chemotherapy led to a rapid and severe clinical deterioration. Cerebrospinal fluid testing detected a high viral load of JCPyV. The patient underwent a brain biopsy, which revealed inflammatory changes consistent with the pathological changes of PML. This case highlights a critical diagnostic challenge and underscores the need for a high index of suspicion for PML in any immunocompromised patient with new-onset neurological symptoms, regardless of whether they have commenced new immunosuppressive therapy. It also serves as a poignant reminder of the potential for chemotherapy to exacerbate an underlying viral pathology.

## 1 Introduction

Progressive multifocal leukoencephalopathy (PML) is a devastating central nervous system (CNS) disorder caused by the opportunistic reactivation of the JC polyomavirus (JCPyV) (1). JCPyV, a subclinical infection virus in a large portion of the adult population, typically remains asymptomatic until severe immunosuppression occurs (2, 3). While PML is most commonly associated with Acquired Immune Deficiency Syndrome (AIDS), its incidence has risen in patients with hematological malignancies, particularly those undergoing intensive immunochemotherapy (2). Recent studies have further elucidated the activity of JCPyV in lymphoma patients (4), suggesting a complex interplay between viral reactivation and lymphoproliferative disorders (5–7). In non-HIV immunocompromised populations, such as those with lymphoma, the incidence of PML is estimated at 8.3 per 100,000 person-years for non-Hodgkin lymphoma patients, with mortality rates often exceeding 50% (8). The presentation of PML can mimic other CNS pathologies, including primary CNS lymphoma (PCNSL) or metastatic lymphoma, due to overlapping clinical and radiological features (2, 9). This diagnostic overlap poses a significant clinical challenge, as the treatments for these two conditions are fundamentally opposed: immunosuppressive chemotherapy for lymphoma versus immune reconstitution for PML (10, 11).

We report a unique case of a patient with relapsed lymphoma who developed progressive neurological symptoms consistent with PML prior to the initiation of any new chemotherapy. This stands in contrast to the majority of documented cases, where PML is diagnosed following the administration of immunomodulatory agents (12, 13). The patient’s condition rapidly deteriorated following the start of Rituximab-Gemcitabine-Dexamethasone-Cisplatin (R-GDP) chemotherapy, revealing the profound impact of immunosuppressive agents on a nascent viral disease process. This case underscores the importance of considering PML as a key differential diagnosis in immunocompromised patients, regardless of recent treatment history.

This case underscores two critical clinical takeaways: first, lymphoma relapse itself can drive sufficient endogenous immune dysfunction to trigger PML as a primary neurological manifestation. Second, it serves as an important warning against the premature use of intensive immunochemotherapy in immunocompromised patients with new neurological symptoms before PML has been definitively ruled out.

## 2 Case presentation

### 2.1 Past medical history and initial presentation

The patient had a complex immunological history. Ten years prior, he underwent a splenectomy for thrombocytopenia, which was suspected to be immune-mediated. Six years prior to presentation, he was diagnosed with B-cell Lymphoma, which was treated with surgery and standard cytotoxic chemotherapy. He tolerated the treatment well and achieved complete response, with no signs of recurrent or systemic disease thereafter. The patient was not receiving any long-term maintenance therapy, such as rituximab. Importantly, serological testing confirmed the patient was negative for HIV.

Initial Symptoms (Time Zero): Four months prior to the final diagnosis, the patient began to exhibit right-hand tremor, progressive right-sided limb weakness, slurred speech, and delayed responsiveness.

### 2.2 Initial diagnostic workup

**Figure 1:**
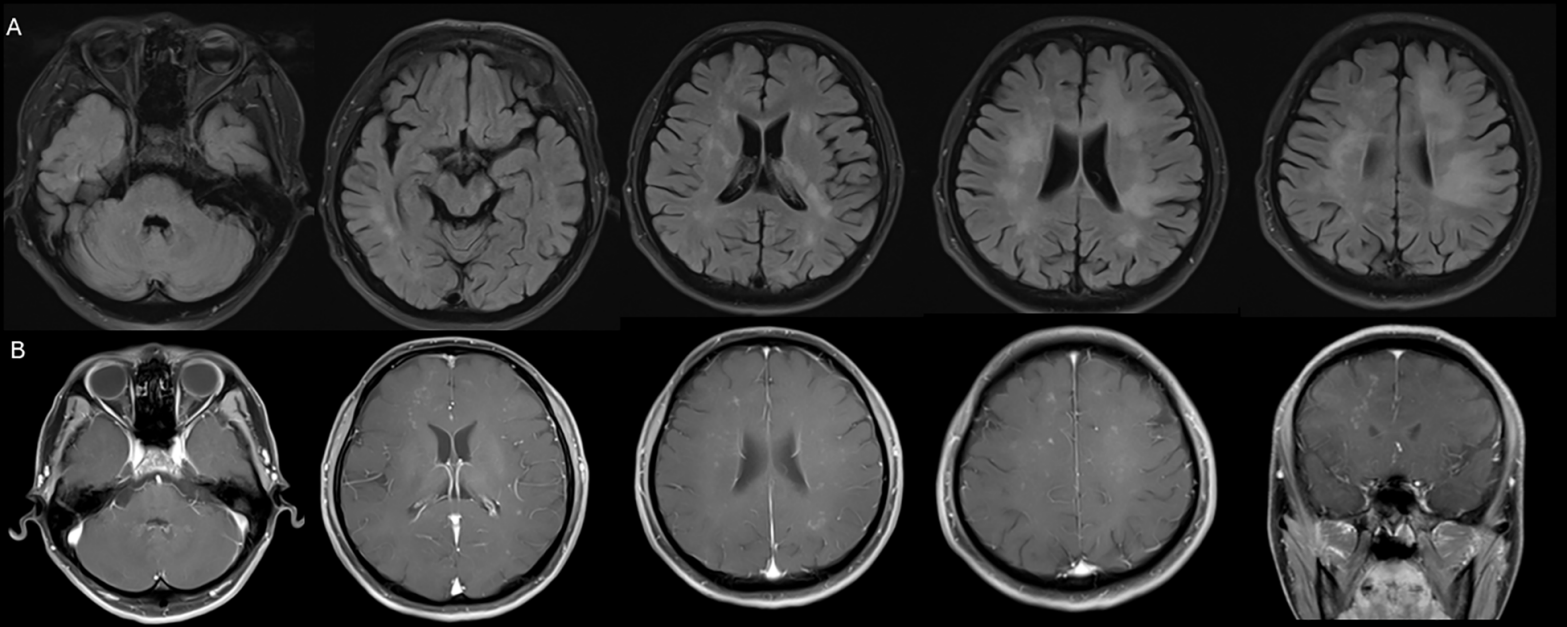
Neuroimaging studies of the patient. **(A)** Multiple patchy T2-FLAIR hyperintensities are noted within the bilateral cerebellar hemispheres, corpus callosum, pons, bilateral fronto-temporo-parieto-occipital lobes, thalamus, basal ganglia, corona radiata, and centrum semiovale. **(B)** On postcontrast sequences, multiple punctate enhancing foci are demonstrated within these regions.

The patient sought care at a local hospital. A brain Magnetic Resonance Imaging (MRI) revealed multiple patchy T2-FLAIR hyperintensities affecting the bilateral cerebellar hemispheres, corpus callosum, pons, bilateral fronto-temporo-parieto-occipital lobes, thalamus, basal ganglia, corona radiata, and centrum semiovale (**Figure 1A**). On post-contrast sequences, these lesions showed multiple punctate enhancing foci (**Figure 1B**). These findings raised initial suspicion of CNS lymphoma infiltration.

A lumbar puncture was performed. Cerebrospinal fluid (CSF) analysis showed a leukocyte count of 2 × 10^6^/L, glucose of 3.03 mmol/L, and protein of 547.7 mg/L. Crucially, CSF quantitative PCR (qPCR) testing detected a high viral load of JCPyV (768 copies/mL). JCPyV DNA was quantified using a real-time qPCR assay targeting the Large T-antigen gene region. Despite this finding confirming active PML, the symptoms were primarily attributed to potential CNS lymphoma infiltration, and the patient received only supportive and antiviral treatments, though symptoms continued to worsen.

### 2.3 Lymphoma relapse and treatment course

One month after symptom onset: A PET-CT scan confirmed systemic lymphoma relapse, demonstrating high FDG uptake in mesenteric lymph nodes and possible infiltration in the left inguinal lymph nodes. The scan also revealed multiple patchy, slightly low-density lesions in the white matter, consistent with PML, which did not exhibit significant FDG uptake. Based on the diagnosis of relapsed lymphoma, the patient was started on the R-GDP chemotherapy regimen (Rituximab, Gemcitabine, Dexamethasone, Cisplatin).

Following the first cycle of chemotherapy, his condition progressively deteriorated, leading to an inability to walk unassisted. Following a second cycle (two months after onset), his speech and comprehension further declined. Following a third cycle (three months after onset), the patient developed a fever (peaking at 38 °C) and experienced a focal seizure characterized by right-gaze deviation and head turning. An Electroencephalogram (EEG) was performed and showed non-specific diffuse background slowing, consistent with severe diffuse encephalopathy.

### 2.4 Final outcome and immunological findings

**Figure 2:**
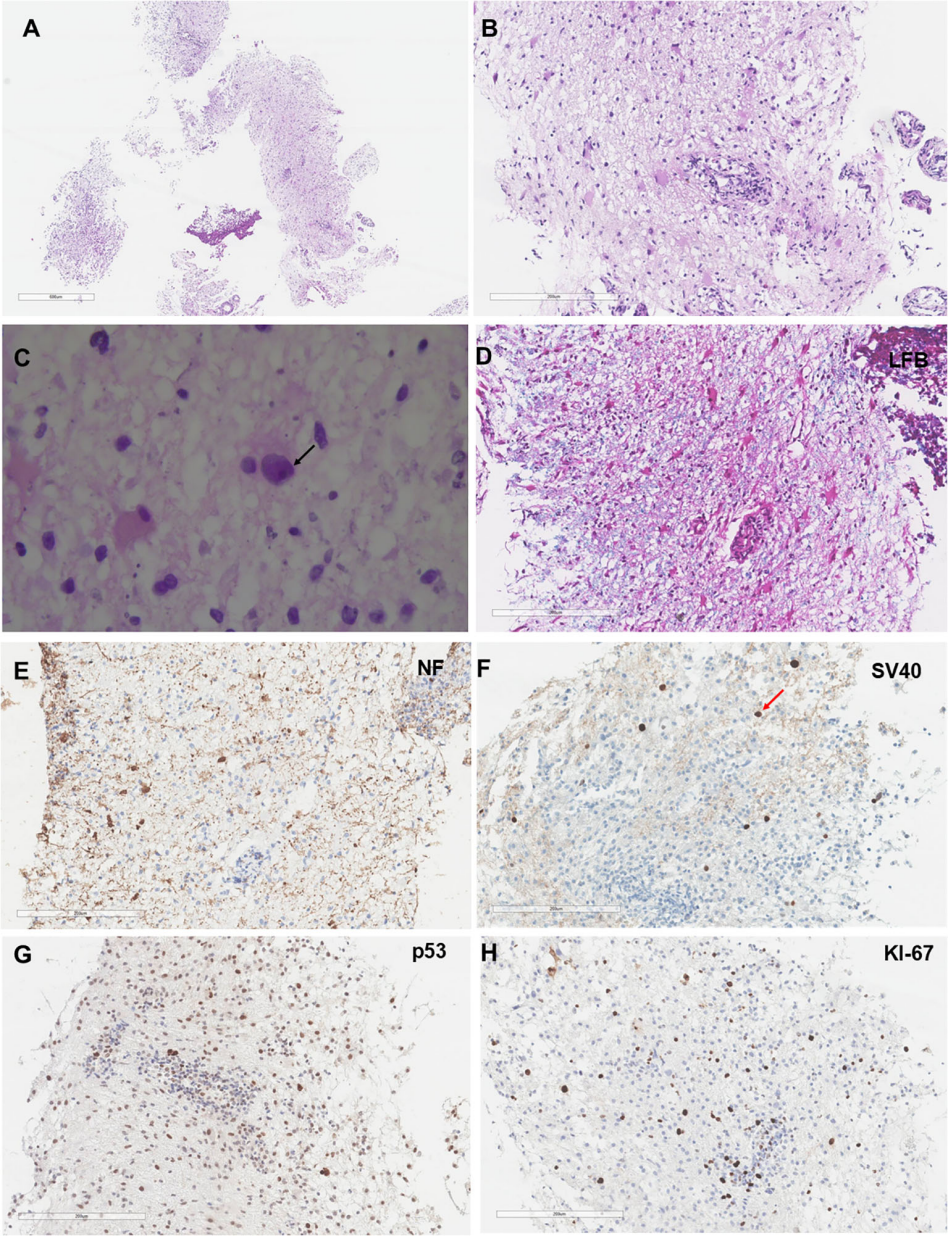
Histological features of the cerebral biopsy specimen from the patient. **(A, B)** Hematoxylin and eosin-stained sections reveal enlarged oligodendrocytes nucleus, and perivascular lymphoplasmacytic cuffing. **(C)** Oligodendrocytes contain 5- to 7-µm amphophilic intranuclear viral inclusions (black arrow). **(D)** LFB+HE staining confirms extensive demyelination in the white matter. **(E)** Neurofilament (NF) immunostaining demonstrates relative preservation of axons. **(F)** SV40 immunoperoxidase staining shows positive (dark brown/black) nuclear staining in oligodendrocytes (red arrow), indicating active polyomavirus replication. **(G, H)** Immunohistochemistry shows that the SV40-infected atypical glial cells exhibit concurrent immunoreactivity for both p53 and Ki-67.

The patient subsequently underwent a brain biopsy, which revealed inflammatory changes (**Figure 2**). Microscopically, lymphocyte, phagocyte and microglia were infiltrated in the cerebral tissue (**Figures 2A, B**). A high-power microscopic image showed inclusion bodies in the oligodendrocyte (**Figure 2C**). Immunohistochemistry and special stains demonstrated demyelination and viral inclusion. Luxol fast blue (LFB) and NF showed the demyelinated lesions (**Figures 2D, E**). Infected oligodendrocytes positive for SV40 (**Figure 2F**), and virally infected cells were positive for P53 and Ki-67 (**Figures 2G, H**). *In situ* hybridization for Epstein-Barr virus-encoded small RNA was negative. Correlated with MRI findings, the overall histological profile confirmed a diagnosis of PML. Blood routine tests at that time showed a lymphocyte percentage of 16%, with an absolute value within the normal range. Lymphocyte subset analysis indicated a significantly decreased B-lymphocyte count (CD19+ at 0.43 cells/µL, ratio 0.03%). In contrast, T-lymphocyte subsets (CD4+ and CD8+) showed normal counts and ratios, with a CD4/CD8 ratio of 2.23. CSF analysis showed a leukocyte count of 2 × 10^6^/L, and CSF qPCR testing detected a high viral load of JCPyV (6319 copies/mL). Flow cytometry analysis of both blood and CSF was normal, showing no evidence of malignant cell infiltration.

Treatment with pembrolizumab was initiated at a dose of 2 mg/kg every three weeks, with the aim of enhancing antiviral T-cell immunity. After one month of pembrolizumab treatment, the patient’s neurological condition stabilized, with no further progression of limb weakness or speech deficits.

## 3 Discussion

This case provides an unusual presentation that challenges the typical understanding of PML pathogenesis in hematological malignancies (10). The most compelling aspect is the onset and virological confirmation of PML (768 copies/mL of JCPyV DNA) before the patient received any cycles of the new R-GDP chemotherapy regimen (9, 10). This contrasts sharply with the majority of reported cases where PML is a complication following the administration of immunomodulatory agents (9). This unique timeline strongly suggests that the patient’s relapsed lymphoma had already caused a state of significant underlying immune dysfunction -a prolonged, endogenous immune dysfunction - that was sufficient to trigger JCPyV reactivation on its own. A retrospective study on PML in patients with hematological cancer found the median time from cancer diagnosis to PML diagnosis was 48.5 months, supporting the concept of a long-term risk (14).

Lymphoma recurrence can manifest in diverse extranodal sites beyond the central nervous system. Skull and calvarial involvement, though uncommon, may present as osteolytic lesions or scalp masses, sometimes as the first sign of relapse (15). Although our patient did not exhibit such findings, clinicians should remain vigilant for signs of skeletal involvement in lymphoma patients with neurological symptoms, as these may indicate disseminated disease and influence both diagnostic and therapeutic strategies.

The patient’s initial symptoms and brain imaging findings presented a classic diagnostic dilemma. Both PML and CNS lymphoma can present with progressive neurological deficits such as hemiparesis and aphasia (9). The MRI findings of multiple abnormal enhancing signals were consistent with both diagnoses (16). The detection of a high JCPyV viral load in the CSF was a critical finding that should have raised immediate suspicion for PML, as this is the gold standard for diagnosis even in cases with negative brain biopsies. Advanced imaging processing techniques are increasingly utilized to improve diagnostic accuracy in intracranial lesions. Although not yet specifically validated for PML versus CNS lymphoma, radiomic signatures could potentially aid early differential diagnosis and monitoring of treatment response, reducing reliance on invasive biopsy in ambiguous cases (17).

Surveillance for lymphoma recurrence in remission patients typically involves clinical follow-up every 3–6 months for the first 5 years, with history, physical examination, and laboratory tests including lactate dehydrogenase (LDH) and complete blood count (18). Clinical suspicion for relapses should be heightened by B-symptoms (fever, night sweats, weight loss), elevated LDH, new lymphadenopathy, or subacute neurological deficits. In such scenarios, prompt systemic staging (PET-CT) and targeted neurological evaluation are warranted.

Based on this case and existing literature, we propose the following diagnostic and therapeutic approach:

1. Initial evaluation: Detailed neurological examination, contrast-enhanced brain MRI, and systemic staging (PET-CT).
2. Early CSF analysis: Include cell count, protein, glucose, cytology, flow cytometry, and quantitative JCPyV PCR.
3. If JCPyV detected and clinical/radiological features compatible with PML:

Withhold intensive immunosuppressive chemotherapy.

Prioritize immune reconstitution strategies (discontinuation of immunosuppressants, consideration of checkpoint inhibitors such as pembrolizumab, or other emerging therapies).

Monitor viral load and clinical status serially.

4. If JCPyV is negative and imaging suggests lymphoma:

Proceed with CNS-directed biopsy if feasible, followed by tailored immunochemotherapy.

5. Serial follow-up: Multimodal monitoring (clinical, MRI, CSF) every 1–3 months initially, with treatment escalation only after PML stabilization or exclusion.

The patient’s lymphocyte subset analysis provides a unique immunological snapshot that is highly instructive. The profound depletion of B-lymphocytes (CD19+ ratio 0.03%) is a key finding. B-cells are known to act as a potential reservoir for JCPyV and may play a direct role in its dissemination to the central nervous system (11). Therefore, the patient’s B-cell deficiency, likely related to the relapsed lymphoma rather than solely the prior treatment, may have contributed to an immune shift favoring JCPyV progression.

Even more striking is the finding that the patient’s T-lymphocyte counts (CD4+ and CD8+) and the CD4/CD8 ratio were within the normal range at the time of diagnosis. Traditionally, PML is thought to develop in the setting of severe cellular immunity deficiency, particularly involving T-cells (2). However, there are documented cases of PML occurring in patients with relatively preserved CD4+ T-cell counts (19). This suggests that T-cell function, rather than just T-cell count, is the critical determinant in controlling JCPyV (20). Recent research suggests that large B-cell lymphomas can leave a persistent “immune scar” that impairs T-cell function and adaptive immunity for years after treatment, even if cell counts appear normal (9). This concept of a “dysfunctional immune phenotype” provides a powerful explanation for why PML could develop in this patient despite normal T-cell counts (21). The patient’s underlying lymphoma relapse and long-term immunodeficiency likely created a state where T-cells were functionally compromised and unable to mount an effective antiviral response, a condition further exacerbated by the B-cell depletion.

The subsequent clinical course, marked by rapid and severe deterioration after the initiation of R-GDP chemotherapy, provides a crucial lesson. The R-GDP regimen includes two potent immunosuppressants: rituximab and dexamethasone. Rituximab is an anti-CD20 monoclonal antibody that selectively depletes B-cells (22) (11), while also having a negative impact on T-cell function (23). Studies have shown that rituximab use is associated with a significantly increased risk of PML, with one study reporting a 19.9-fold increased risk among non-HIV-infected patients with chronic lymphocytic leukemia (10). Dexamethasone, a powerful glucocorticoid, further suppresses T-cell function and broader cellular immunity (24). The combination of these two agents provided a “double hit” to an already weakened immune system, creating an ideal environment for the fulminant progression of PML, which was already present before treatment started. This clinical observation strongly supports the hypothesis that the chemotherapy, rather than causing the PML, acted as a “fatal accelerator.”

The brain biopsy confirmed inflammation-lymphocyte and phagocyte infiltration-consistent with a diagnosis of CNS- Immune Reconstitution Inflammatory Syndrome in the setting of PML. However, the classification of this inflammation requires critical reconsideration. Our patient’s condition worsened dramatically following the initiation of intensive immunosuppression (R-GDP), accompanied by an explosive, nearly eight-fold increase in JCPyV viral load (from 768 to 6319 copies/mL). This clinical trajectory directly opposes the mechanism of immune reconstitution. We therefore contend that the patient experienced an Atypical, Treatment-Accelerated Inflammatory Flare. The pathological inflammation observed (infiltration of lymphocytes/phagocytes) is not the result of immune reconstitution but rather a severe, localized cytopathic reaction to the massive, accelerated viral lysis and subsequent myelin destruction induced by the overwhelming immunosuppression from the R-GDP regimen.

This case is a powerful testament to the complexity of diagnosing neurological symptoms in immunocompromised patients. It demonstrates that PML can be a primary manifestation of lymphoma relapse due to inherent immune dysfunction, a finding that is extremely rare in the literature (10). Furthermore, it serves as a critical warning against the premature use of intensive immunochemotherapy in such patients without first ruling out PML (9). A low diagnostic threshold for PML, coupled with early and thorough CSF JCPyV testing, is paramount to avoid potentially life-threatening misdiagnoses and therapeutic missteps.

## 4 Conclusion

This case report details an extraordinary presentation of PML as a primary neurological manifestation of relapsed lymphoma, occurring before the commencement of new systemic therapy. The rapid clinical deterioration following R-GDP chemotherapy highlights the profound risk of administering immunosuppressive treatments in a patient with a pre-existing, subclinical viral CNS infection. Early and accurate diagnosis through CSF analysis is crucial to guide appropriate management and prevent further clinical decline.

## Data Availability

The original contributions presented in the study are included in the article/supplementary material. Further inquiries can be directed to the corresponding author.
